# Stationary phase expression of the arginine biosynthetic operon *argCBH *in *Escherichia coli*

**DOI:** 10.1186/1471-2180-6-14

**Published:** 2006-02-22

**Authors:** Jeevaka P Weerasinghe, Tao Dong, Michael R Schertzberg, Mark G Kirchhof, Yuan Sun, Herb E Schellhorn

**Affiliations:** 1 McMaster University, Department of Biology, Life Sciences Building, Rm. 218, 1280 Main Street West, Hamilton, ON, Canada, L8S 4K1

## Abstract

**Background:**

Arginine biosynthesis in *Escherichia coli *is elevated in response to nutrient limitation, stress or arginine restriction. Though control of the pathway in response to arginine limitation is largely modulated by the ArgR repressor, other factors may be involved in increased stationary phase and stress expression.

**Results:**

In this study, we report that expression of the *argCBH *operon is induced in stationary phase cultures and is reduced in strains possessing a mutation in *rpoS*, which encodes an alternative sigma factor. Using strains carrying defined *argR*, and *rpoS *mutations, we evaluated the relative contributions of these two regulators to the expression of *argH *using operon-*lacZ *fusions. While ArgR was the main factor responsible for modulating expression of *argCBH*, RpoS was also required for full expression of this biosynthetic operon at low arginine concentrations (below 60 μM L-arginine), a level at which growth of an arginine auxotroph was limited by arginine. When the *argCBH *operon was fully de-repressed (arginine limited), levels of expression were only one third of those observed in Δ*argR *mutants, indicating that the *argCBH *operon is partially repressed by ArgR even in the absence of arginine. In addition, *argCBH *expression was 30-fold higher in Δ*argR *mutants relative to levels found in wild type, fully-repressed strains, and this expression was independent of RpoS.

**Conclusion:**

The results of this study indicate that both derepression and positive control by RpoS are required for full control of arginine biosynthesis in stationary phase cultures of *E. coli*.

## Background

The biosynthesis and/or scavenging of arginine are important during host colonization by uropathogenic *Escherichia coli*. In urine, expression of the *E. coli argCBH *operon and *artJ*, encoding a periplasmic transporter, increases more than 10 fold [1] and 18 fold [2], respectively. Synthesis of arginine is likely required during infection as the concentration of arginine found in urine is below that necessary to support maximal growth of *E. coli *[1]. Consistent with these data, infection challenge in a murine model with *E. coli *strains carrying mutations in the *argC *gene results in impaired proliferation in the kidney [1]. In enteropathogenic *E. coli *[3] arginine synthesis and transport, together with arginine decarboxylase (encoded by *adiA*), are important components of the cell's acid resistance repertoire [3-5].

Under nutrient-limiting conditions, *E. coli *can potentially utilize arginine as both a carbon and a nitrogen source [6]. Arginine is a precursor for the synthesis of polyamines, putrescine and spermidine, which may reduce oxidative damage to proteins and DNA [7,9]. In addition, in phosphate-starved cells, aerobic metabolism of arginine may be an important physiological adaptation that is intimately associated with cell survival [10]. Since arginine contains 11% of the cell's nitrogen in stationary phase [11], biosynthesis of this amino acid is likely important under sub-optimal conditions.

Arginine is synthesized by a complex biosynthetic pathway consisting of several operons and unlinked genes that are controlled by ArgR [12] which represses by binding to a conserved ARG box [13,14] to overlap with RNA polymerase binding sites. Maximum derepression occurs in the absence of arginine [12]. In contrast to most amino acid biosynthetic genes, the expression of the *arg *biosynthetic genes increases briefly during diauxic growth arrest [15]. As this stress is similar to that imposed by nutrient limitation, it is plausible that stationary phase regulators participate in control of arginine biosynthesis. Several enzymes required for arginine catabolism are controlled by RpoS, an alternative stationary phase sigma factor [16], including those encoded by *astD *[11,17], and *cstC *(*astC*) [11,18]. Many of the members of the large RpoS regulon are specifically expressed during the transition to stationary phase growth [19,20]. Although clearly required for virulence in *Salmonella *[21], the role of RpoS in the pathogenesis of *E. coli *is equivocal. RpoS controls many functions that contribute to host adaptation, including osmotic stress [22] and acid challenge [3]. In *E. coli*, however, RpoS mutants are not impaired in colonization of the urinary tract [23] or gastrointestinal tract [24] in animal models. There are several different alleles of RpoS found in natural populations of *E. coli *[25], and it is thus possible that RpoS regulation may be strain specific.

In a previous study, we identified many independent RpoS-dependent operon fusions [26]. Two of these mapped to the *argCBH *operon and were of particular interest because, unlike other RpoS-regulated functions that we identified, this operon, when mutated, rendered the cell auxotrophic. In this study, we have employed these fusions as probes to examine regulatory controls on transcription of the *argCBH *operon to identify how this key biosynthetic pathway is activated.

## Results

### Characterization of *argCBH *operon fusions

**Figure 1:**
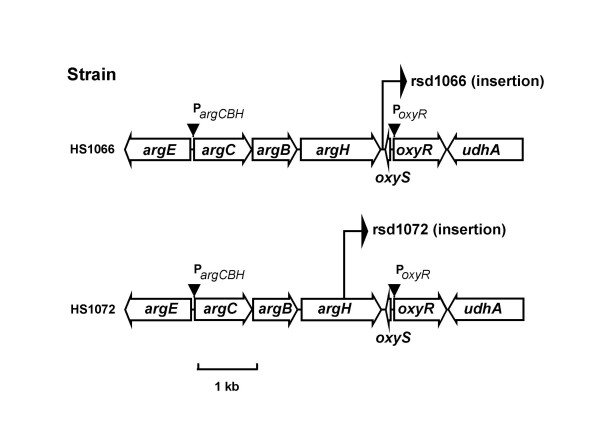
Location of operon fusions in strains used in this study. Arrows indicate the direction of transcription of genes.

**Figure 2:**
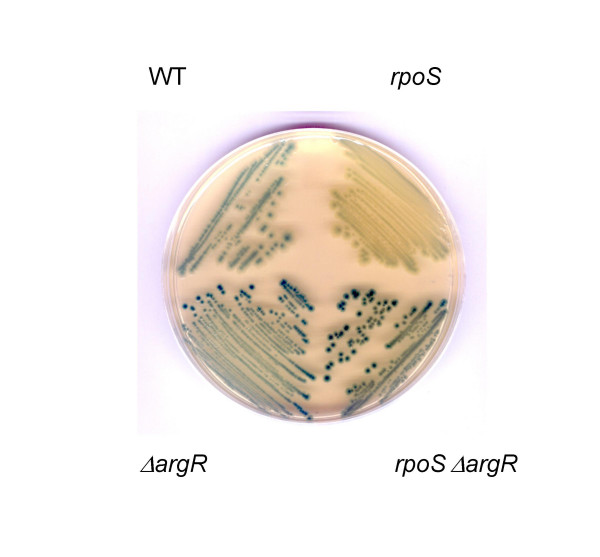
Expression of *argCBH-lacZ *in WT, Δ*rpoS*, Δ*argR *and Δ*rpoS*Δ*argR *strains on LB plates containing X-Gal.

In a large mutational screen, we isolated over 100 RpoS dependent operon-*lacZ *fusions [26,27], many of which were not known to require RpoS for expression. Two of the isolated fusion mutations mapped to the *argCBH *operon (Fig. 1) and were clearly RpoS-dependent on indicator plates (Fig. 2). While both fusions were in *argH*; one was intragenic and, as expected, rendered the cell auxotrophic for arginine (*rsd*1072) and the other fusion (*rsd*1066) was located between the *argH *coding sequence and a predicted transcriptional terminator (Fig. 1). Strains carrying the *rsd*1066 fusion do not have an arginine auxotrophic phenotype (data not shown).

Since ArgR is a known regulator of *argCBH *[12], we constructed combinatorial *argR *and *rpoS *mutants to determine the relative contributions of each regulator to the expression of the operon. Introduction of a deletion of *argR *into a strain with an *argCBH-lacZ *fusion resulted in high expression with or without RpoS (Fig. 2). It is likely that RpoS-dependent expression of *argCBH *in the Δ*argR *strains may be masked by strong derepression as a consequence of loss of ArgR.

### Growth phase dependence of *argCBH *expression

To test if the expression of the *argCBH *operon is induced upon entry into stationary phase, expression of the operon was assayed in rich media. During entry into stationary phase, expression of *argCBH *increased 7-fold in wild type cultures, but only exhibited 3-fold increase in the Δ*rpoS *mutant (Table 1). This indicates that *argCBH *expression is not only growth phase-dependent but also is affected by loss of RpoS. Expression of *argCBH *expression was 14-fold higher in Δ*argR *mutants than in wild type strains and in these mutants, was independent of RpoS (Table 1).

### Effect of exogenous arginine on *argCBH *expression

**Figure 3:**
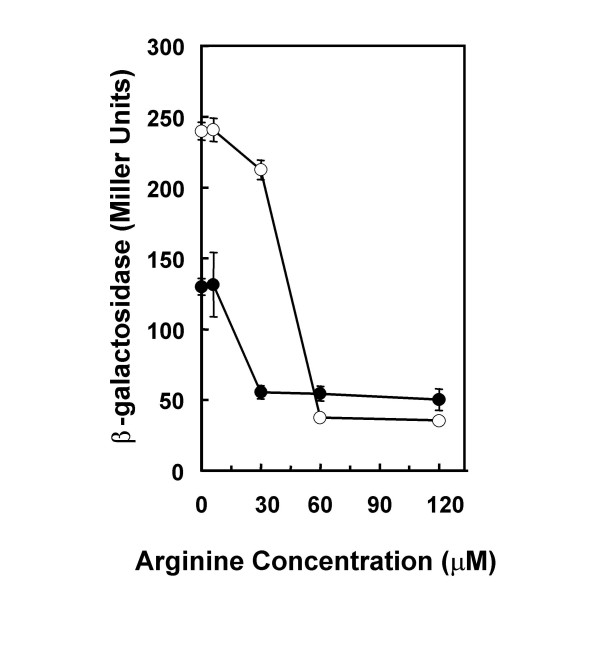
Expression of *argCBH *in isogenic wild type (○) and *rpoS *mutant (●) strains in glucose minimal media supplemented with exogenous arginine. Expression was tested in exponential phase (OD_600 _= 0.3)

RpoS is likely an important factor in amino acid scavenging in stationary phase [28]. Amino acid biosynthesis offers an alternative to the scavenging strategy. It is possible that the RpoS effect on *argCBH *expression is more pronounced when arginine becomes limiting, as might be the case in stationary phase. To test this, expression of the operon was assessed in exponential phase cultures grown in minimal media supplemented with various concentrations of L-arginine. In strain HS1066 and its *rpoS *mutant derivative, HS1066p, *argCBH *expression was found to be inversely proportional to arginine concentration at arginine concentrations less than 60 μM (Fig. 3). Arginine biosynthetic genes, including *argCBH*, are normally induced in response to arginine limitation [29]. However, in exponential phase cultures, *argCBH *expression was RpoS dependent only at arginine concentrations below 30 μM (Fig. 3). In stationary phase, *argCBH *expression was 2-fold RpoS-dependent at all arginine concentrations tested (data not shown).

Surprisingly, Δ*argR *mutants were impaired in growth on minimal media even when supplemented with arginine, strongly suggesting that these strains possessed an additional unidentified nutritional requirement. Intermediates in the arginine biosynthetic pathway are also precursors in other pathways. For example, carbamoylphosphate, the product of carbamoylphosphate synthetase (encoded by the bi-cistronic operon *carAB*) is not only a precursor of arginine but is also required for pyrimidine biosynthesis [30]. Therefore, it is possible that the derepression of arginine biosynthesis may deplete carbamoylphosphate required for synthesis of pyrimidine thus rendering Δ*argR *mutants auxotrophic for pyrimidine. To test this, we examined the growth of Δ*argR *mutants on minimal media supplemented with pyrimidines relative to *argR*^+ ^strains. As shown in Table 2, the growth deficiency of Δ*argR *mutants could be completely suppressed by the addition of pyrimidines (growth of Δ*argR *and wild type strains were equivalent in the presence of added pyrimidines). We also compared the generation time of *argR*^+ ^and Δ*argR *strains on minimal media with or without pyrimidines, and examined the average colony size by plating the cells on minimal media plates. As shown in Table 3, the generation time of Δ*argR *was much greater than that of the *argR*^+ ^strain, and this growth impairment could be remedied by addition of pyrimidines into the media. The growth requirement was not absolute as some residual growth was observed in the Δ*argR *strain. Reduced growth could be due to either a uniform slow growth among cells or by the selection of suppressor mutants which could overcome impairment by acquiring advantageous mutations. The diameters of Δ*argR *deletion colonies were only one half that of *argR*^+ ^colonies indicating ArgR is required for robust growth in minimal media. Furthermore, the colony morphology and size of all colonies were uniform, consistent with the idea that growth results were a consequence of poor growth of *argR *deletion mutants in general and were not due to suppression by selected mutants. To ensure that this was not a strain-specific phenotype, the Δ*argR *mutation was transduced and tested in another common laboratory strain, MG1655 and, as expected, the resultant transductants were similarly found to be pyrimidine limited (data not shown). To exclude the possibility that an uncharacterized mutation linked to the original operon fusion mutation was responsible for the pyrimidine phenotype, we tested an independently constructed *argR *deletion mutant from the Nara KO collection [31]. This strain was similarly impaired in pyrimidine synthesis (data not shown).

We further examined the pyrimidine requirement by transforming an *argR *deletion mutant with an *argR*-containing plasmid clone from the ASKA collection [31]. Colony size of the ArgR-complemented *argR *deletion mutant was more than twice that of the control strain after two days growth on minimal media (0.76 ± 0.03 mm vs. 0.34 ± 0.03 mm).

### Effect of an *astCADBE *operon deletion on expression of *argCBH*

**Figure 4:**
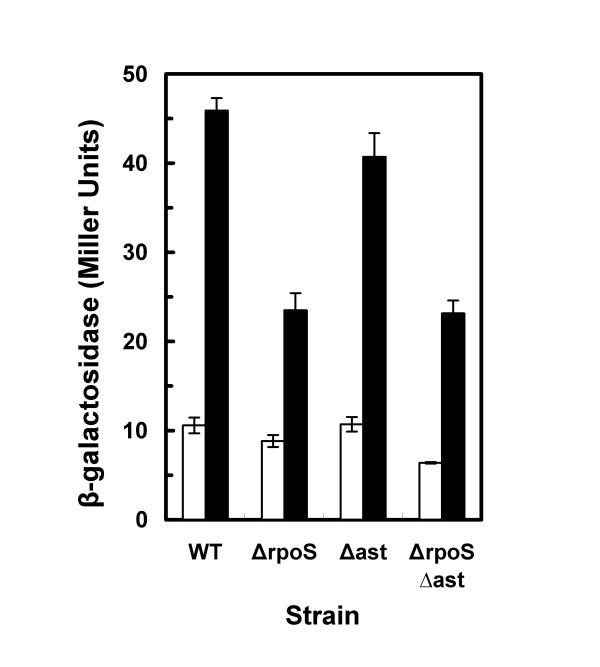
Effect of *astCADBE *depleted arginine on *argCBH *expression in wild type, *ΔrpoS, Δast *,and *ΔrpoS Δast *strains. Strains were grown in triplicate in LB rich media. Samples were taken in exponential phase (□, OD_600 _= 0.3) and stationary phase (■, OD_600 _= 1.5), and β-glactosidase activity was assayed as described in Methods.

The *astCADBE *operon, encoding a set of enzymes responsible in the arginine succinyltransferase (AST) pathway, is RpoS-dependent and can be induced in nitrogen limited environment especially when arginine is present [11]. It is possible that RpoS affects *argCBH *expression through AST-mediated depletion of intracellular arginine resulting in increased derepression by ArgR. To test this possibility, an *astCADBE *deletion mutant was constructed in strains containing the *argCBH-lacZ *fusion, and the resultant mutant was assayed in rich media. In exponential phase there is not much expression difference in all these strains. However, in stationary phase, the expression of *argCBH *in *Δast *mutation strain was nearly 2-fold (an increase of 18 units) higher than that in *ΔrpoS Δast *double mutation strains (Fig. 4). Thus, the RpoS-dependence of *argCBH *in *Δast *deletion background is consistent with results obtained using AST^+ ^strains, and it is unlikely that RpoS-modulated *argCBH *expression is indirectly induced by arginine catabolism through the AST pathway. However, the expression of *argCBH *in *Δast *mutant was slightly lower (10% difference) than in the AST^+ ^strain, indicating that metabolism of arginine through the AST pathway may have a slight overall effect on *argCBH *expression, probably through modulation of ArgR.

### The effect of exogenous arginine on growth

**Figure 5:**
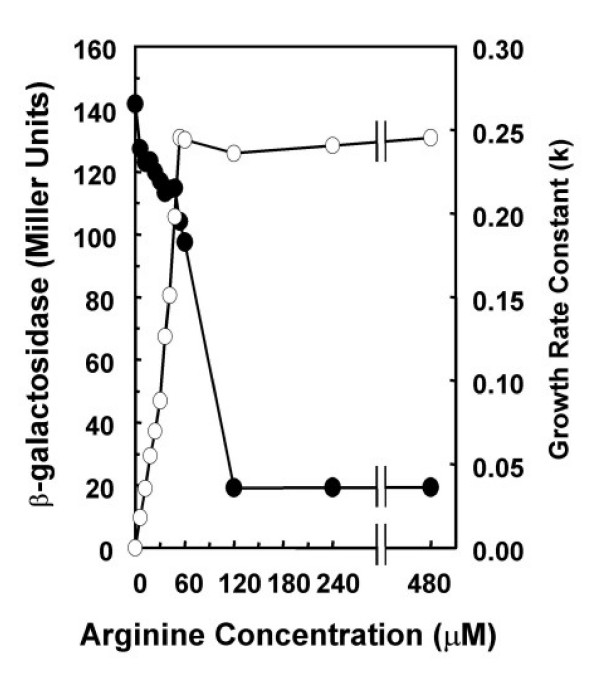
Effect of exogenous arginine on growth and *argCBH *expression. Growth rate and *argCBH *expression were determined using an arginine auxotrophic strain, HS1072 and an arginine prototrophic strain HS1066, respectively, at the indicated arginine concentrations. Overnight cultures were grown with appropriate antibiotics, sub-cultured into minimal media and maintained in exponential phase for at least 8 generations prior to the start of the experiment.

Since strains carrying a mutation in *argH *are auxotrophic for arginine (HS1072 this study), these strains could be used to establish the concentration at which this key amino acid becomes growth limiting. Similarly, because the operon fusion in strain HS1066 does not render the cell arginine auxotrophic but is nonetheless ArgR dependent, this strain can be used in parallel to assess ArgR-dependent activation of the operon during arginine restriction. Growth of *E. coli *was impaired at arginine concentrations less than 60 μM: below this level growth rate was proportional to concentration of the limiting amino acid. Growth yield was similarly affected (data not shown). Derepression of *argCBH *expression was found to occur at concentrations below 60 μM and, as expected, was inversely related to arginine concentration (Fig. 5). Limitation by the availability of intracellular arginine may account for the reduced growth observed and, through ArgR derepression, may also be partially responsible for the increased expression of *argH*. Together, these data indicate that *argCBH *regulation is tightly coupled to arginine availability. At 60 μM, even though the cultures were not arginine-limited with respect to growth, the levels of *argCBH *were five fold higher than in fully repressed cells. This suggests that, in nutrient-starved cells, *argCBH *derepression would occur prior to potential growth restriction by arginine limitation ensuring maximum growth yield.

### Northern analysis of *argH *expression

**Figure 6:**
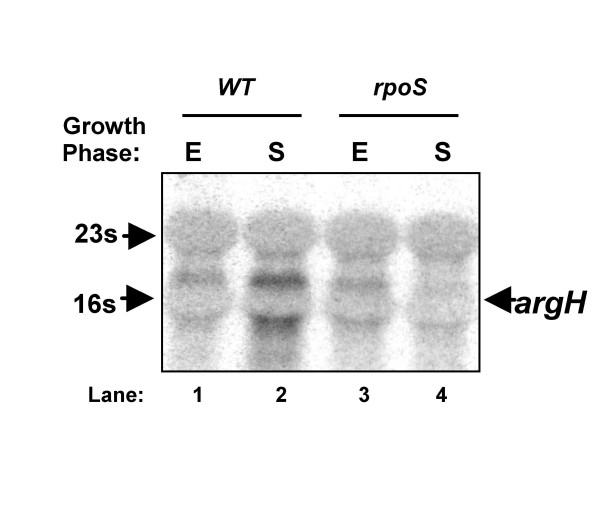
RpoS dependent expression of *argH *in exponential and stationary phase determined by Northern analysis. RNA was extracted from cultures grown in LB to OD_600 _= 0.3 for exponential phase (E) and OD_600 _= 1.5 for stationary phase (S) using the hot phenol method (as described in Methods). 5 μg of RNA was loaded in each lane. Signal intensity was quantified by densitometry and normalized to an arbitrary value of 10 for expression of the operon in stationary phase in the wild type strain.

Although plate expression assays indicate that *argH *expression is clearly RpoS-dependent (Fig. 2), the results of reporter gene expression studies showed that RpoS dependence was only 2-fold, somewhat less than that found for most RpoS regulated genes [26]. To resolve this discrepancy, we independently assessed expression of *argH *in wild type and *rpoS *mutant cultures by Northern analysis (Fig. 6). Consistent with reporter gene studies on plates, expression of *argH *was much higher in stationary phase cultures of the wild type strain than in the isogenic *rpoS *mutant and was much lower in exponential phase samples of both cultures. The hybridizing *argH *transcript co-migrated with the 16S RNA leading to some interference (Fig. 6). These results, in conjunction with the reporter fusion studies, indicate that expression of *argH *is both RpoS and stationary phase dependent in a wild type strain and is thus similar to many other RpoS-dependent genes [26].

### Expression of Argininosuccinate lyase, the product of *argH*

To determine if RpoS and ArgR modulation of *argH *expression results in changes in the level of the encoded enzyme, we assayed exponential phase cultures of strains deficient in the expression of these regulators for argininosuccinate lyase activity. This was tested by growing cultures in minimal media at suboptimal levels since the RpoS effect was most pronounced when cells were slightly starved for arginine (Fig. 3).

The specific ArgH activity in WT was about 4 fold higher than that in *rpoS*^- ^strain, while it was about 25 fold higher in *argR*^- ^strain compared with the WT (Table 4). These results, together the *lacZ *expression and Northern data, supports the idea modulation of *argH *expression by RpoS is reflected at the level of protein synthesis.

## Discussion

In this study, we examined RpoS-dependent control of *argCBH *expression, and its modulation by ArgR and external arginine in *Escherichia coli*. Using independently-isolated mutants carrying operon fusions in different positions within the *argCBH *region, we conducted expression studies of this biosynthetic operon by assaying reporter gene fusions and by examining *argH *transcript levels in both a wild type strain and an *rpoS *mutant by Northern analysis. Since ArgR also regulates this operon [12], we evaluated the relative contributions of both ArgR and RpoS to its expression by constructing appropriate double and single null mutants.

RpoS regulates many genes that play important roles in stress resistance and energy metabolism [16], but a subset of these RpoS-dependent genes including *gabP *[26], *proP *[32], *proU *[33], *gadAB *[34] and *ldcC *[35] aid in amino acid transport and utilization. In a previous genetic screen for RpoS-dependent genes [26], we identified one mutant that was auxotrophic for arginine and carried a mutation that mapped to the terminal gene member of the *argCBH *operon. To the best of our knowledge, this is the only RpoS-modulated gene known which, when mutated, renders the cell auxotrophic. As such, examining the regulation of this operon may offer unique insight into RpoS-controlled stationary phase physiology.

Our data showing that stationary phase expression of the *argCBH *operon is affected by RpoS does not reveal whether this effect is direct or indirect. In fact for many members of the RpoS regulon such information has not been established. Indirect regulation is known to be operant for at least some members, including *gadA *and *gadB*, two glutamate decarboxylases that are among the most highly RpoS-dependent genes based on microarray analysis [27]. The expression of these genes depends on GadX, a regulator whose growth phase dependent increase expression requires RpoS [36].

RpoS can regulate its operon members directly (e.g. *osmY *[37]) or indirectly (e.g. *gadW *by the RpoS-dependent GadX regulator [38]). As RpoS dependence of *argCBH *was not observed in Δ*argR *mutants, ArgR appears to be necessary for RpoS-modulated expression of the operon. There are at least two mechanisms that could explain the increase in expression in stationary phase. In the first, as RpoS activates expression of its large regulon, depletion of intracellular arginine may result as a consequence of *de novo *synthesis of stationary phase proteins. This may derepress the arginine biosynthetic pathway, resulting in an increase in *argCBH *expression. Alternatively, increased catabolism through the RpoS-dependent AST pathway lower intracellular arginine and might also result in de-repression of the operon. The latter explanation, however, seems unlikely in view of the fact that RpoS modulation of *argCBH *is not affected by deletion of the AST operon (this study).

Using the arginine auxotroph (HS1072), we found that *E. coli *becomes growth limited at arginine concentrations below 60 μM. As derepression also occurs at these concentrations (this study), it appears that expression of the biosynthetic pathway is closely coupled to the biosynthetic need for arginine. As the concentration of arginine in LB media is about 60 μM [9], it is possible that our results could be partially explained by depletion of arginine during growth in late exponential phase. However, as supplementation with exogenous arginine did not markedly reduce stationary phase induction of the *argCBH *operon, it is likely that other mechanisms, including control by RpoS, ensure that this operon continues to be expressed under nutrient limited conditions.

Surprisingly, maximum levels of *argCBH *expression in the wild type strain were only one third of those found in an isogenic Δ*argR *mutant suggesting that ArgR can be an active repressor even in the absence of exogenous arginine. This may be due to the fact that, even under starvation conditions, synthesized endogenous arginine can be an effective co-repressor. Such control may be physiologically necessary as carbamoylphosphate, an arginine precursor, is also required for pyrimidine biosynthesis. Balancing these two pathways, arginine and pyrimidine biosynthesis, under nutrient-limited conditions, is likely an important physiological imperative, as complete derepression of arginine biosynthesis, by deletion of *argR*, causes cells to develop a partial requirement for exogenous pyrimidine (this study). The need for *de novo *arginine synthesis therefore appears to be balanced against other biosynthetic requirements of the cell. he multiple controls on *carAB*, including availability of arginine, pyrimidines as well purines [39] ensure that the synthesis of these macromolecule precursors is balanced in actively growing cells.

Why might arginine biosynthesis be stationary phase dependent? It is well established that nutrient scavenging is an important survival mechanism in starved cultures [40]. Arginine in particular is likely to be an important metabolite in stationary phase cultures for several reasons. As arginine represents 11 percent of the cells total nitrogen [11], it is potentially an important nitrogen reservoir for starving populations. Arginine is also a potential precursor for the biosynthesis of polyamines which stabilize and condense DNA during senescence [41] and protect it against oxidative damage [7,8,42]. Finally, as *de novo *protein synthesis in non-growing stationary phase cells is required for the expression of stationary phase adaptive proteins, this may impose a significant biosynthetic demand upon the cell both because there are many such proteins produced (see [16] for review) and because some of these are expressed to extraordinarily high levels. For example, Dps, a highly RpoS-dependent DNA binding protein [43], is almost undetectable in exponential phase, but accumulates to 200,000 molecules per cell in stationary phase (approx. 5% of total cellular protein) [44]. Many genes are induced upon entry into stationary phase and it is likely that this creates a high demand for amino acids for *de novo *protein synthesis. The up-regulation of amino acid biosynthetic operons such as *argCBH *may provide a means to satisfy this demand in addition to nutrient scavenging mechanisms including arginine transport which is also a key factor in maintaining high arginine levels in stationary phase cultures [45].

**Figure 7:**
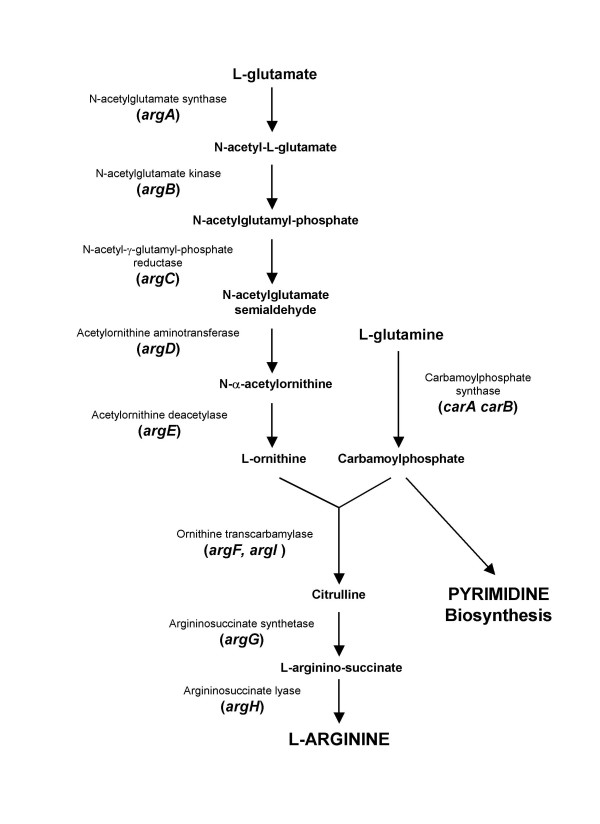
The arginine biosynthetic pathway. Note that ArgF and ArgI are ornithine transcarbamylase, while the *carAB *operon encodes subunits of carbamoylphosphate synthase. Adapted from EcoCyc: Encyclopedia of *Escherichia coli *Genes and Metabolism .

Biosynthetic regulons, particularly those required for amino acid biosynthesis, are often controlled by a transcriptional repressor (e.g. TrpR and ArgR). Though *argCBH *operon is modulated by RpoS in stationary phase cultures the dynamic range of this control was small in relation to that exerted by ArgR. Interestingly, another member of the ArgR regulon, a*stC*, a member of the *astCADBE *operon, is RpoS-dependent to a similar degree [17]. These observations, in conjunction with the results of this study, suggest that RpoS plays an important role in coordinately regulating arginine metabolism in stationary phase. Such control is likely effective because of the nature of the pathway. ArgA-mediated synthesis of N-acetylglutamate is the first committed step in the arginine biosynthetic pathway and is controlled by 1) cumulative feedback inhibition by arginine and 2) ArgR at the level of transcription [12]. Since ArgC and ArgB catalyze early steps in this pathway and ArgH catalyzes the final biosynthetic reaction (Fig. 7), it is likely that control of these key steps by ArgR/RpoS modulates the entire arginine biosynthetic pathway.

While our studies employed a non-pathogenic *E. coli *K-12 strain, the results of this study may have relevance for *E. coli *pathogenesis. For example, though urine is a good growth media for uropathogenic *E. coli *[46], low concentration of several key nutrients, including arginine [46] and iron [2], can be limiting. As both transport [2] and biosynthesis [1] of arginine are required for maximum growth in urine and in minimal media containing restrictive levels of arginine, control of the functions for the metabolism of this amino acid are likely critical for urovirulence. As RpoS has now been implicated in both control of biosynthesis (this study) and catabolism of arginine [18], it will be useful, in future studies, to establish the relative importance of these metabolic functions in pathogenesis.

## Conclusion

In summary, *argCBH *expression is clearly controlled by a finely balanced mechanism mediated by two signals: 1) a general nutrient stress signal mediated, in part, by RpoS and, 2) well known specific control through arginine-dependent modulation of the ArgR repressor.

## Methods

### Bacterial strains, phage, and plasmids

All strains used are *E. coli *K-12 derivatives. The bacterial strains, phage and plasmids used in this study are listed in Table 5.

### Media and chemicals

All chemicals were supplied by either Sigma Chemical or Gibco BRL. Cultures were routinely grown in Luria-Bertani (LB) media and in M9 minimal media [47]. The antibiotics used were ampicillin (100 μg ml^-1^), chloramphenicol (25 μg ml^-1^), kanamycin (50 μg ml^-1^), tetracycline (15 μg ml^-1^), and streptomycin (100 μg ml^-1^).

### Growth conditions

All cultures were grown in triplicate from independently isolated colonies. Cell growth was monitored spectrophotometrically (Novaspec^® ^II spectrophotometer, Pharmacia LKB Biochrom Ltd., Cambridge, England) by measuring optical density at 600 nm (OD_600_). Expression studies in rich media were conducted using cultures maintained in early exponential phase (OD_600 _of < 0.3) in antibiotic-free LB media for at least 8 generations, prior to the start of the experiment. Sub-cultures with a starting OD_600 _of 0.01 were grown in LB at 37°C and agitated at 200 rpm.

To quantify the RpoS dependence of *argCBH *expression in relation to exogenous arginine concentration in minimal media, overnight minimal media cultures (0.4% glucose) were inoculated from well-isolated colonies on minimal media plates (0.2% glucose) and grown with appropriate antibiotics. To ensure complete repression of arginine biosynthesis, the overnight cultures were supplemented with 230 μM L-arginine. The cultures were diluted (1 in 1000) and maintained in early exponential phase (as described above) prior to the start of experiments in antibiotic-free minimal media supplemented with 230 μM L-arginine. At an OD_600 _of 0.3, cultures were placed on ice for 2 min and then centrifuged for 10 min at 4000 × g at room temperature. The supernatant was decanted and the resulting cell pellets washed twice with arginine-free minimal media to remove remaining exogenous arginine. The arginine-free cell pellets were re-suspended in minimal media to the same optical density as collected. A series of sub-cultures with a starting OD_600 _of 0.05 were made into minimal media supplemented with various concentrations of exogenous L-arginine. The sub-cultures were incubated at 37°C and agitated at 200 rpm. For each concentration of L-arginine, samples were taken in mid-exponential phase (OD_600 _of 0.3) and assayed for β-galactosidase activity

### Enzyme Assays

β-galactosidase activity was assayed as previously described by Miller [47]. ONPG was used as the substrate, and activity was expressed in Miller units [47]. All cultures were grown in triplicate from independent colony isolates (biological replicates) and all assays were performed in duplicate.

Argininosuccinate lyase (ASL) activity was determined by measuring the absorbance of fumarate hydrolyzed from argininosuccinate at OD_240 nm _[48]. Cell extracts were prepared by sonication [49]. The reaction mixtures containing 10 ug/ml protein, 1.0 mM argininosuccinate in 50 mM potassium phosphate buffer (pH7.5) were monitored photometrically at OD_240 nm_. One unit of ASL activity corresponds to 1 nmole L-argininosuccinate hydrolysized per min at pH 7.5 and 37°C.

### Construction of Δ*argR *and Δ*astCADBE *deletion mutants

The one-step chromosomal gene inactivation procedure of Datsenko and Wanner [50] was used to generate precise Δ*argR *and Δ*astCADBE *deletion mutations. The mutation was transduced [47] into other strains using P1vir transduction (see Table 5).

The pKD3 plasmid was used as a template to amplify the PCR fragments used for replacement of the *argR *and *astCADBE *target genes. Primers were designed such that the resulting PCR product includes the Cm^r ^cassette from pKD3 flanked by sequences adjacent to the target reading frame in the MG1655 chromosome. The PCR primers used for *argR *were: forward 5'-**CAATAATGTTG TATCAACCACCATATCGGGTGACTT**GTGTAGGCTGAAGCTGCTTC-3' and reverse 5'-**ACATTTTCCCCGCCGTCAGAAACGACGGGGCAGAGA**CATATGA ATATCCTCCTTAG-3'; primers used for creating *astCADBE *deletion were: forward 5'-**ACTTAATACCCGCAGAATGATTTCTGCGGGTAAGTA**GTGTAGG CTGGAGCTGCTTC-3' and reverse 5'-**CATATAAATAACGAATTATTTACTGTA GAGGTCGCT**CATATGAATATCCTCCTTAG-3'. The bold text corresponds to target gene flanking sequences, and normal text represents DNA sequences of the Cm^r ^cassette. The deletion generated the entire target coding sequence.

Incorporation of the Cm^r ^cassette into the MG1655 chromosome was confirmed by PCR using genomic DNA as a template and confirmational primers. The confirmational forward primers used were the forward primers (as described above). The reverse confirmational primer for *argR *was 5'-TGTCGCA GTAAAACGCACTA-3', for *astCADBE *was 5'-TTATACGCAAGGCGACAAGG-3'.

All primers were synthesized by MOBIXLab, McMaster University (Hamilton, ON).

### RNA Isolation and Northern Analyses

RNA was isolated from cultures grown in LB using the hot phenol method [51]. Primers to the *argH *gene (5'-CGGTTCAAACAATTCAACGA-3' and 5'-GCAGCTTTTTGCCTAACTGG-3') were used to PCR-amplify a DNA probe for hybridization studies to examine stationary phase and RpoS dependence of *argH *expression. RNA samples were prepared, separated by electrophoresis and hybridized as previously described [26]. Probes were prepared and radioactively labeled by PCR as described in [52]. Densitometric analysis of the bands was performed using a Storm^® ^(Amersham Biosciences, Inc., Baie d'Urfe, QC) gel and blotting imaging system with ImageQuant™ v 5.2 (Amersham Biosciences, Inc.).

## Authors' contributions

J.P.W. and T.D performed most of the experiments and, with M.R.S., wrote the first draft. M.R.S. also performed the Northern analysis. M.G.K. aided in the construction of the *argR *deletion strains and helped in the design of several experiments. S.Y. designed and conducted additional arginine supplementation experiments. H.E.S was the principal investigator and supervised the project.

## Figures and Tables

**Table 1:** Expression of the *argCBH *operon in rich media. Wildtype, Δ*rpoS *and Δ*argR *strains harboring the rsd1066 operon *lacZ*-fusion to the *argCBH *operon were grown in LB media and assayed for β-galactosidase activity in exponential phase (OD_600 _= 0.3) and stationary phase (OD_600 _= 1.5) as described in Methods. All reported activities are the average of three independent determinations.

**Strain**	**Genotype**	**β-Galactosidase (Miller Units)**	
		
		**Exponential**	**Stationary**
HS1066	WT	5.6 ± 0.34	38.4 ± 0.97
HS1066p	Δ*rpoS*	6.1 ± 0.26	18.8 ± 0.15
HS2404	Δ*argR*	469 ± 18	553 ± 33
HS2405	Δ*rpoS *Δ*argR*	486 ± 32	712 ± 35

**Table 2:** Effect of exogenous supplemented pyrimidines on growth of Δ*argR *strains. Overnight cultures were grown in minimal media with exogenously-supplemented L-arginine (230 μM). Cultures were washed in arginine-free minimal media and replica-plated onto minimal plates supplemented with exogenous cytosine, thymine and uracil. Growth was scored after overnight incubation at 37°C. "-" no growth on the plates after 24 hours incubation

**Strain**	**Genotype**	**Media**		
		
		**LB**	**M9 + G**	**M9 + G + P**
HS1066	WT	+	+	+
HS1066p	Δ*rpoS*	+	+	+
HS2404	Δ*argR*	+	-	+
HS2405	Δ*rpoS*Δ*argR*	+	-	+

G, Glucose

P, Pyrimidine- Cytosine, Uracil and Thymine added at 20 μg per ml

**Table 3:** Effect of Δ*argR *on culture growth in minimal media. Overnight cultures in LB were washed in minimal salts 3 times and subcultured at 1:1000 dilution into minimal media and grown at 37°C, 200 rpm, and samples were plated on minimal media to determine the average colony size.

**Strain**	**Genotype**	**Media**	**Generation Time (h)**	**Average Colony (mm)**
HS1066	*argR*^+^	M9	1.31 ± 0.12	1.21 ± 0.02
HS2404	Δ*argR*	M9	2.14 ± 0.28	0.74 ± 0.02
HS1066	*argR*^+^	M9 + P	1.21 ± 0.07	1.30 ± 0.03
HS2404	Δ*argR*	M9 + P	1.27 ± 0.23	0.73 ± 0.02

P, Pyrimidine- Cytosine, Uracil and Thymine added at 20 μg per ml

**Table 4:** Specific argininosuccinate lyase (ASL) activity in exponential culture of WT, *rpoS*^-^, and Δ*argR *strains. Cultures were grown in M9 minimal media supplemented with 30 μM L-arginine and harvested at OD_600 nm _= 0.3. Cell extracts were assayed as described in the Methods section. ASL enzyme assay values were corrected for the low background levels of non-specific activity in the *argH*^- ^mutant.

**Strain**	**Genotype**	**Argininosuccinate lyase (U/mg protein)**	**Per cent of wild type**
GC4468	WT	6.50 ± 0.083	100
GC122	*rpoS*^-^	1.53 ± 0.86	24
HS2404	Δ*argR*	159.6 ± 22.0	2,455
HS1072	*argH-lacZ*	0	0

**Table 5:** *E. coli *strains and bacteriophage used in this study.

**Strains**	**Genotype**	**Source/Reference**
MG1655	Prototrophic *E. coli *K-12, F^- ^λ^-^*rph*	CGSC, Yale University
GC4468	Δ*lacU169 rpsL*	
GC122	as GC4468 but *rpoS13*::Tn*10*	[49]
HS1066	as GC4468 but *argH*^+^-λp*lac*Mu53[Φ(*argH-lacZ*)66](*rsd*1066)	[26]
HS1066p	as HS1066 but *rpoS13::*Tn*10*	[26]
HS1072	as GC4468 but *argH*^-^-λp*lac*Mu53[Φ(*argH-lacZ*)72](*rsd*1072)	[26]
HS1072p	as HS1072 but *rpoS13::*Tn*10*	[26]
HS2401	as MG1655 but Δ*argR::*cat	This study
HS2402	as GC4468 but Δ*argR::*cat	P1(HS2401)×GC4468→Cm^R^
HS2403	as GC122 but Δ*argR::*cat	P1(HS2401)×GC122→Cm^R^
HS2404	as HS1066 but Δ*argR::*cat	P1(HS2401)×HS1066→Cm^R^
HS2405	as HS1066p but Δ*argR::*cat	P1(HS2401)×HS1066p→Cm^R^
HS2406	as HS1072 but Δ*argR::*cat	P1(HS2401)×HS1072→Cm^R^
HS2407	as HS1072p but Δ*argR::*cat	P1(HS2401)×HS1072p→Cm^R^
HS3006	As HS1066 but Δ*astCADBE::cat*	This study
HS3006p	As HS3006 but *rpoS13::*Tn*10*	P1(HS3006)×HS1066p→Cm^R^
**B) Phage**		
P1*vir*	generalized transducing phage	Laboratory stock
**C) Plasmids**		
pKD3	Template plasmid for gene disruption, *cat *is flanked by FRT sites.	[50]
pKD46	λ Red recombinase expression plasmid under control of an *araC*-P_*araB *_inducible promoter.	[50]
pArgR	Derivative of pCA24N	H. Mori [31]
